# Alternative kynurenic acid synthesis routes studied in the rat cerebellum

**DOI:** 10.3389/fncel.2015.00178

**Published:** 2015-05-18

**Authors:** Tonali Blanco Ayala, Rafael Lugo Huitrón, Liliana Carmona Aparicio, Daniela Ramírez Ortega, Dinora González Esquivel, José Pedraza Chaverrí, Gonzalo Pérez de la Cruz, Camilo Ríos, Robert Schwarcz, Verónica Pérez de la Cruz

**Affiliations:** ^1^Departamento de Neuroquímica, Instituto Nacional de Neurología y Neurocirugía Manuel Velasco Suárez, S.S.A.México D.F., Mexico; ^2^Laboratorio de Neuroquímica, Instituto Nacional de Pediatría, S.S.A.México D.F., Mexico; ^3^Facultad de Química, Departamento de Biología, Universidad Nacional Autónoma de MéxicoMéxico D.F., Mexico; ^4^Facultad de Ciencias, Departmento de Matemáticas, Universidad Nacional Autónoma de MéxicoMéxico D.F., Mexico; ^5^Maryland Psychiatric Research Center, Department of Psychiatry, University of Maryland School of MedicineBaltimore, MD, USA

**Keywords:** D-amino acid oxidase, kynurenine, microdialysis, oxidative stress, reactive oxygen species

## Abstract

Kynurenic acid (KYNA), an astrocyte-derived, endogenous antagonist of α7 nicotinic acetylcholine and excitatory amino acid receptors, regulates glutamatergic, GABAergic, cholinergic and dopaminergic neurotransmission in several regions of the rodent brain. Synthesis of KYNA in the brain and elsewhere is generally attributed to the enzymatic conversion of L-kynurenine (L-KYN) by kynurenine aminotransferases (KATs). However, alternative routes, including KYNA formation from D-kynurenine (D-KYN) by D-amino acid oxidase (DAAO) and the direct transformation of kynurenine to KYNA by reactive oxygen species (ROS), have been demonstrated in the rat brain. Using the rat cerebellum, a region of low KAT activity and high DAAO activity, the present experiments were designed to examine KYNA production from L-KYN or D-KYN by KAT and DAAO, respectively, and to investigate the effect of ROS on KYNA synthesis. In chemical combinatorial systems, both L-KYN and D-KYN interacted directly with peroxynitrite (ONOO^−^) and hydroxyl radicals (OH•), resulting in the formation of KYNA. In tissue homogenates, the non-specific KAT inhibitor aminooxyacetic acid (AOAA; 1 mM) reduced KYNA production from L-KYN and D-KYN by 85.1 ± 1.7% and 27.1 ± 4.5%, respectively. Addition of DAAO inhibitors (benzoic acid, kojic acid or 3-methylpyrazole-5-carboxylic acid; 5 μM each) attenuated KYNA formation from L-KYN and D-KYN by ~35% and ~66%, respectively. ONOO^−^ (25 μM) potentiated KYNA production from both L-KYN and D-KYN, and these effects were reduced by DAAO inhibition. AOAA attenuated KYNA production from L-KYN + ONOO^−^ but not from D-KYN + ONOO^−^. *In vivo*, extracellular KYNA levels increased rapidly after perfusion of ONOO^−^ and, more prominently, after subsequent perfusion with L-KYN or D-KYN (100 μM). Taken together, these results suggest that different mechanisms are involved in KYNA production in the rat cerebellum, and that, specifically, DAAO and ROS can function as alternative routes for KYNA production.

## Introduction

In the mammalian brain, the tryptophan metabolite kynurenic acid (KYNA) functions as an endogenous antagonist of the α7 nicotinic acetylcholine receptor (α7nAChR; Hilmas et al., 2001) and the N-methyl-D-aspartate receptor (NMDAR; Kessler et al., 1989; Alkondon et al., 2011). KYNA, which is also a ligand of the G protein-coupled receptor GPR35 (Wang et al., 2006) and can activate the aryl hydrocarbon receptor (DiNatale et al., 2010), is considered a neuromodulator since fluctuations in its endogenous levels bi-directionally influence extracellular concentrations of glutamate, dopamine and γ-aminobutyric acid (GABA) levels in the rat brain (Carpenedo et al., 2001; Rassoulpour et al., 2005; Amori et al., 2009; Wu et al., 2010; Pocivavsek et al., 2011; Beggiato et al., 2014), and reductions in KYNA formation result in increased levels of extracellular acetylcholine (Zmarowski et al., 2009). Notably, increases in cerebral KYNA levels, which are seen in the aged brain (Moroni et al., 1988; Gramsbergen et al., 1992; Heyes et al., 1992; Kepplinger et al., 2005) and in several major neurological and psychiatric diseases (Baran et al., 1999; Schwarcz et al., 2001; Guidetti et al., 2004; Kepplinger et al., 2005; Sathyasaikumar et al., 2011), have been suggested to be causally related to cognitive impairments (Wonodi and Schwarcz, 2010; Pocivavsek et al., 2012, 2014).

In the brain as elsewhere, KYNA synthesis is attributed to several distinct kynurenine aminotransferases (KATs), which catalyze the irreversible transamination of L-kynurenine (L-KYN) to KYNA (Okuno et al., 1991; Guidetti et al., 2007a; Han et al., 2010). Of these enzymes, KAT II, which is preferentially contained in astrocytes (Guidetti et al., 2007b), has received most attention since it appears to be responsible for the rapid mobilization of newly produced KYNA (Schwarcz et al., 2012). However, alternative routes of KYNA production exist under physiological conditions. For example, KYNA can be formed from D-kynurenine (D-KYN) through oxidative deamination by D-amino acid oxidase (DAAO; Loh and Berg, 1971; Ishii et al., 2010), as demonstrated in the brain and in peripheral tissues of mice, rats and rabbits (Mason and Berg, 1952; Loh and Berg, 1971; Fukushima et al., 2009; Wang et al., 2012) and, recently, in human brain tissue (Pérez-de la Cruz et al., 2012). However, KATs also recognize D-KYN as a substrate and can catalyze the *de novo* formation of KYNA from D-KYN in the brain *in vivo* (Pérez-de la Cruz et al., 2012).

Neosynthesis of KYNA can also involve the transamination of L-tryptophan by tryptophan-2-oxoglutarate aminotransferase (Hardeland, 2008). Thus, the enolic form of the primary reaction product, indole-3-pyruvic acid, is highly susceptible to reactive oxygen species (ROS) and readily undergoes pyrrole ring cleavage by interaction with oxygen intermediaries. The transiently formed product then spontaneously cyclizes to generate KYNA. L-KYN, too, is easily oxidized and can be converted to KYNA in the presence of hydrogen peroxide (H_2_O_2_), a process that is substantially enhanced by horseradish peroxidase (Zsizsik and Hardeland, 2001b). In biological systems, too, KYNA formation can result from direct reactions of either indole-3-pyruvic acid or KYN with ROS. Examples include KYNA synthesis in several rat organs after incubation with indole-3-pyruvic acid under conditions that are conducive to the generation of free radicals (ascorbate/Fe/H_2_O_2_) (Politi et al., 1991), and KYNA production from L-KYN in homogenates of *Lingulodinium polyedrum* exposed to light and various ROS generators (Zsizsik and Hardeland, 2001a, 2002).

The present study was designed to examine the various routes of KYNA neosynthesis from L-KYN and D-KYN in parallel. Using the rat cerebellum, which was selected on the basis of its high DAAO content and relatively low KAT activity (Baran and Schwarcz, 1993; Horiike et al., 1994; Moreno et al., 1999; Verrall et al., 2007), we also compared KYNA formation in the presence or absence of ROS. Our results demonstrate that *de novo* KYNA formation can involve different mechanisms, and that ROS should be considered a viable alternative for KYNA production from both L-KYN and D-KYN under physiological and, possibly, pathological conditions.

## Materials and Methods

### Animals

Adult, male Wistar rats (280–320 g), obtained from the vivarium of the National Autonomous University of Mexico (Mexico City), were used for this study. The animals were housed five per cage in acrylic cages and provided with a standard commercial rat diet (Laboratory rodent diet 5001, PMI Feeds Inc., Richmond, IN, USA) and water *ad libitum*. All rats were housed in the same room under identical environmental conditions, i.e., temperature (25 ± 3°C), humidity (50 ± 10%) and lighting (12 h light/dark cycles).

Animals were killed by decapitation, and their tissues were immediately dissected out on ice and preserved at −70°C. All procedures with animals were carried out according to the National Institutes of Health Guide for the Care and Use of Laboratory Animals, and the local guidelines on the ethical use of animals from the Health Ministry of Mexico. All efforts were made to minimize animal suffering during the study.

### Materials

KYNA, L-KYN, D-KYN, dimethylsulfoxide (DMSO), DL-penicillamine, diethylenetriaminepentaacetic acid (DTPA), H_2_O_2_, ethylenediaminetetraacetic acid (EDTA), 3-methylpyrazole-5-carboxylic acid (MPC), kojic acid and benzoic acid were obtained from Sigma Aldrich Company (St. Louis, MO, USA). All other chemicals were of the highest commercially available purity. Solutions were prepared using deionized water obtained from a Milli-RQ (Millipore) purifier system.

### ONOO^−^ Synthesis

ONOO^−^ was synthesized as previously described (Beckman et al., 1994). Five ml of an acidic solution (0.6 M HCl) of H_2_O_2_ (0.7 M) were briefly mixed with 5 ml of 0.6 M KNO_2_ in an ice bath, and the reaction was quenched with 5 ml of ice-cold 1.2 M NaOH. Residual H_2_O_2_ was removed using granular MnO_2_ pre-washed with 1.2 M NaOH, and the reaction mixture was then left overnight at −20°C. The resulting yellow liquid layer on top of the frozen mixture was collected for the experiment immediately before use, and adjusted to a final concentration of 50 μM using Ringer solution (144 mM NaCl, 4.8 mM KCl, 1.2 mM MgSO_4_ and 1.7 mM CaCl_2_, pH 7.2). Concentrations of ONOO^−^ were determined in quartz cuvettes using a molar extinction coefficient of 302 nm = 1670 M^−1^cm^−1^ (Hughes and Nicklin, 1970).

### Chemical Combinatorial Assays

The ability of OH• to produce KYNA in combination with L-KYN or D-KYN was examined using the Fe^3+^-EDTA-H_2_O_2_ system (Halliwell et al., 1987; Floriano-Sánchez et al., 2006). The system contained L-KYN or D-KYN (20 μM each), 0.2 mM ascorbic acid, 0.2 mM FeCl_3_, 0.2 mM EDTA, 1 mM H_2_O_2_ and 20 mM phosphate buffer (pH 7.4) in a final volume of 500 μl. Additional tubes were incubated in the presence of 10% DMSO to evaluate the effect of an OH• scavenger on KYNA production. Samples were incubated for 15 min at room temperature. After incubation, KYNA production was quantified by high performance liquid chromatography (HPLC; see below).

Interactions between the two KYN enantiomers and ONOO^−^ were determined using ONOO^−^ synthetized in our laboratory (Lugo-Huitrón et al., 2011a). Briefly, the reaction mixture (in a final volume of 500 μl in HPLC grade water) consisted of L-KYN or D-KYN (20 μM each) and 25 μM ONOO^−^. In separate tubes, the ONOO^−^ scavenger DL-penicillamine (300 μM) (Floriano-Sánchez et al., 2006); was added to evaluate its effect on KYNA formation. After 15 min of incubation at room temperature, KYNA levels were determined by HPLC.

### *In vitro* Studies with Tissue

Cerebella were dissected out and immediately weighed and frozen on dry ice. Tissues were then homogenized (1:10, w/v) in Krebs buffer (118.5 mM NaCl, 4.75 mM KCl, 1.77 mM CaCl_2_, 1.18 mM MgSO_4_, 12.9 mM NaH_2_PO_4_, 3 mM Na_2_HPO_4_ and 5 mM glucose; pH 7.4). In order to evaluate KYNA production by ONOO^−^, 80 μl of the tissue homogenate were incubated for 2 h at 37°C in the presence of DAAO inhibitors (MPC, benzoic acid or kojic acid) or AOAA. L-KYN or D-KYN (100 μM) were added to the tissue homogenate, and each inhibitor (final concentration: 1 mM) was assessed in the presence or absence of ONOO^−^ (25 μM) in a final volume of 200 μl. After incubation, samples were centrifuged for 10 min at 6,000 × g, and the supernatants were diluted 1:5 (v/v) for KYNA determination.

### Microdialysis

Rats were anesthetized with a mixture of ketamine (80 mg/kg) and xylazine (100 mg/kg) (i.p.) and placed in a stereotaxic frame. A guide cannula was positioned and secured to the skull with stainless steel screws and acrylic dental cement at the following coordinates: AP: 11.0 mm posterior to bregma, L: ±2.0 mm from the midline, V: 4.0 mm below the dura. Three days later, a microdialysis probe (MD-220, membrane length: 2 mm; BASi, West Lafayette, IN, USA) was inserted through the guide cannula to protrude into the cerebellar cortex, and connected to a microperfusion pump set at a speed of 2 μl/min. Microdialysis samples were collected every 30 min. A stable baseline was first established by perfusing Ringer solution (pH 7.4) for 2 h. Production of KYNA from either L-KYN or D-KYN was then assessed by perfusing the bioprecursors, diluted in Ringer solution, for 2 h. The effect of ONOO^−^ was examined by perfusing the compound for 30 min prior to the administration of either L-KYN or D-KYN. After the discontinuation of the experimental interventions, Ringer solution was perfused for an additional 4 h. Animals were then killed by decapitation, and the cerebellum was dissected to confirm the proper placement of the microdialysis probe. Microdialysate samples were diluted as needed and then analyzed directly by HPLC. Data were not corrected for recovery from the microdialysis probe.

### KYNA Analysis

KYNA was measured by HPLC with fluorometric detection. Briefly, 20 μl of the sample (after either *in vitro* incubations or perfusions *in vivo*) were injected onto a 3-μm C_18_ reverse phase column (80 × 4.6 mm; ESA, Chelmsford, MA, USA), and KYNA was isocratically eluted using a mobile phase containing 250 mM of zinc acetate, 50 mM sodium acetate and 3% of acetonitrile (pH adjusted to 6.2 with glacial acetic acid) at a flow rate of 1 ml/min. KYNA was detected fluorimetrically (excitation wavelength: 344 nm, emission wavelength: 398 nm, S200 fluorescence detector; Perkin-Elmer, Waltham, MA, USA). The retention time of KYNA was ~7 min.

### Data Analysis

One-way analysis of variance (ANOVA) followed by Bonferroni’s *post hoc* test was used to analyze the effect of the different treatments used *in vitro*. In microdialysis experiments, the average of five samples collected immediately prior to the administration of test compounds was defined as the baseline value (100%). The effect of either L-KYN or D-KYN, alone or after *in vivo* pretreatment with ONOO^−^, was analyzed by two-way ANOVA with repeated measures followed by *post hoc* tests: a Student’s *t*-test was used to compare two treatments at a specific time point, and a paired Student’s *t*-test was used when comparing the effect of a treatment at two specific times. In all cases, a *P*-value <0.05 was considered significant.

## Results

### KYNA Production by Interaction of L-KYN or D-KYN with OH• and ONOO^−^ in Synthetic Systems

**Figure 1:**
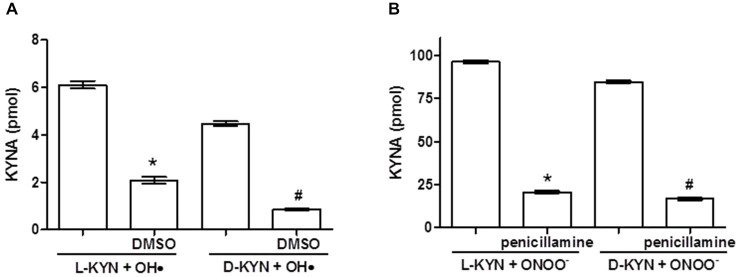
**Incubation of OH• (A) or ONOO^−^ (B) with L-KYN or D-KYN (20 μM each) for 15 min *in vitro* results in the *de novo* production of KYNA in the absence of tissue**. Co-incubation with the radical scavengers DMSO or penicillamine reduces KYNA formation. No KYNA was detected when any of the reagents was incubated alone. See text for further experimental details. Data (mean ± SEM of 6 experiments per group) represent the KYNA concentration in 500 μl. *P < 0.05 vs. L-KYN + OH• or ONOO^−^, ^#^*P* < 0.05 vs. D-KYN + OH• or ONOO^−^ (one-way analysis of variance (ANOVA) followed by Tukey’s *post hoc* test).

Co-incubation of L-KYN or D-KYN (20 μM each) with OH• and ONOO^−^, respectively, resulted in the formation of KYNA *in vitro*. As illustrated in Figure 1, incubation for 15 min led to the *de novo* production of KYNA. KYNA was undetectable in the control solutions containing the enantiomers alone, and no KYNA was measurable when OH• was incubated on its own (not shown). Addition of the OH• scavenger DMSO (10%), which did not contain measurable amounts of KYNA itself, reduced KYNA production from OH• + L-KYN or OH• + D-KYN by 65.8 ± 2.3% and 80.7 ± 1.0%, respectively (Figure 1A). KYNA production was substantially more pronounced following the co-incubation of either L-KYN or D-KYN with ONOO^−^ (25 μM). No KYNA was detectable when ONOO^−^ was incubated on its own (not shown). Addition of the ONOO^−^ scavenger DL-penicillamine (300 μM), which did not contain KYNA on its own, decreased KYNA production from either of the two enantiomers by 78.5 ± 0.7% and 80.1 ± 0.8% for L-KYN and D-KYN, respectively (Figure 1B).

### KAT and DAAO Inhibitors Attenuate KYNA Production from L-KYN and D-KYN in Tissue Homogenate

**Figure 2:**
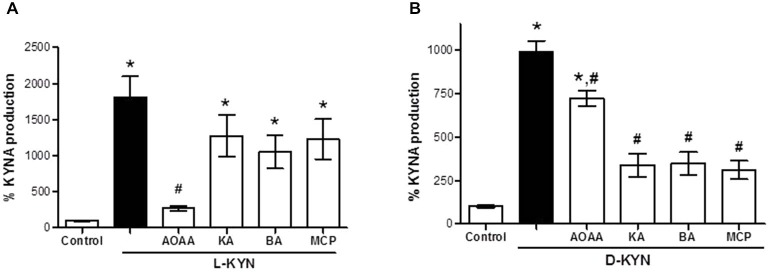
**Effect of KAT and DAAO inhibition on KYNA production from L-KYN or D-KYN (100 μM each) in cerebellar tissue homogenate**. Enzyme inhibitors were used at 1 mM. See text for further experimental details. Data are expressed as a percentage of endogenous tissue levels of KYNA (control; 26.4 ± 2.2 pmoles KYNA/mg protein) and represent the mean ± SEM of 8 experiments per group. *P < 0.05 vs. control, ^#^*P* < 0.05 vs. L-KYN **(A)** or D-KYN **(B)** alone (one-way ANOVA followed by Tukey’s *post hoc* test). AOAA: Aminooxyacetic acid, KA: kojic acid, BA: benzoic acid, MPC: 3-methylpyrazole-5-carboxylic acid.

Incubation of tissue homogenate with L-KYN (Figure 2A) and D-KYN (Figure 2B), respectively, resulted in the *de novo* production of KYNA. The KYNA levels recovered after 2 h incubation with 100 μM of the enantiomers were 18.1 ± 2.9-fold and 9.8 ± 0.7-fold higher, respectively, than basal levels. Incubation in the presence of the KAT inhibitor AOAA (1 mM) reduced KYNA formation from L-KYN and D-KYN by 85.1 ± 1.7% and 27.1 ± 4.5%, respectively, suggesting differences in the mechanisms by which the two enantiomers are converted to KYNA in the cerebellum. In line with this conclusion, the DAAO inhibitors kojic acid, benzoic acid and MPC (all at 1 mM) reduced the production of KYNA from D-KYN by ~66% (Figure 2B) but did not inhibit KYNA formation from L-KYN (Figure 2A).

### ONOO^−^ Potentiates KYNA Production from L-KYN and D-KYN in Tissue Homogenate: Attenuation by KAT and DAAO Inhibitors

**Figure 3:**
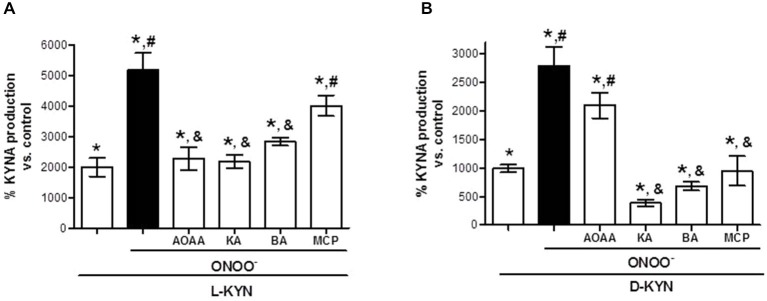
**ONOO^−^ (25 μM) enhances the production of KYNA from L-KYN or D-KYN (100 μM each) in cerebellar tissue homogenate**. The effects of KAT and DAAO inhibitors were tested at 1 mM. See text for further experimental details. Data (mean ± SEM of 8 experiments per group) are expressed as a percentage of endogenous tissue levels of KYNA (control; 26.4 ± 2.2 pmoles/mg protein). *P < 0.05 vs. control, ^#^*P* < 0.05 vs. L-KYN **(A)** or D-KYN **(B)**, ^&^*P* < 0.05 vs. L-KYN + ONOO^−^ **(A)** or D-KYN + ONOO^−^ **(B)** (one-way ANOVA followed by Tukey’s *post hoc* test). AOAA: Aminooxyacetic acid, KA: kojic acid, BA: benzoic acid, MPC: 3-methylpyrazole-5-carboxylic acid.

The addition of ONOO^−^ (25 μM) to tissue homogenate increased KYNA production from both L-KYN and D-KYN (each 100 μM) 2.6 ± 0.3 and 2.8 ± 0.3 times, respectively (Figure 3). Under these conditions, the presence of AOAA (1 mM) attenuated the ONOO^−^-induced potentiation of KYNA formation from L-KYN (Figure 3A) but not from D-KYN (Figure 3B). DAAO inhibitors (all at 1 mM) decreased KYNA production induced by the co-incubation of both L-KYN and D-KYN with ONOO^−^, though the effect of MCP vs. L-KYN + ONOO^−^ did not reach statistical significance. Kojic acid and benzoic acid were particularly effective, reducing the total KYNA generated by the combination of D-KYN + ONOO^−^ by 86.1 ± 2.0% and 75.6 ± 2.7%, respectively.

### Effect of L-KYN and D-KYN on Extracellular KYNA *in vivo*

**Figure 4:**
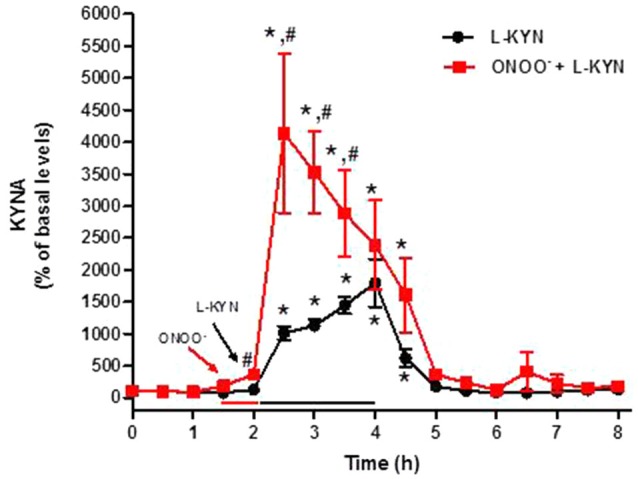
**Effect of reverse dialysis of ONOO^−^ (50 μM) and/or L-KYN on extracellular KYNA production in the rat cerebellum *in vivo***. After a 30 min infusion of ONOO^−^ (red bar), L-KYN (100 μM) was infused for 2 h (black bar; *n* = 6). In separate rats (*n* = 8), L-KYN (100 μM) was applied without pre-treatment. Results (mean ± SEM) are expressed as a percentage of basal values (3.2 ± 0.4 nM). *P < 0.05 vs. baseline, ^#^*P* < 0.05 vs. L-KYN alone (two-way ANOVA followed by paired Student’s *t*-test was used to compare the treatment effect at a specific timepoint vs. baseline; and Student’s *t*-test was used to compare ONOO^−^ + L-KYN vs. L-KYN alone at a specific timepoint).

**Figure 5:**
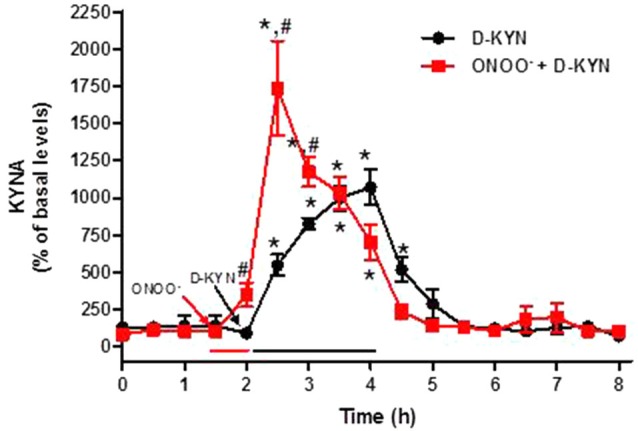
**Effect of reverse dialysis of ONOO^−^ (50 μM) and/or D-KYN on extracellular KYNA production in the rat cerebellum *in vivo***. After a 30 min infusion of ONOO^−^ (red bar), D-KYN (100 μM) was infused for 2 h (black bar; *n* = 8). In separate rats (*n* = 11), D-KYN was applied without pre-treatment. Results (mean ± SEM) are expressed as a percentage of basal values (2.9 ± 0.5 nM). *P < 0.05 vs. baseline, ^#^*P* < 0.01 vs. D-KYN alone (two-way ANOVA followed by paired Student’s *t*-test was used to compare the treatment effect at a specific timepoint vs. baseline; and Student’s *t*-test was used to compare ONOO^−^ + D-KYN vs. D-KYN alone at a specific timepoint).

We next designed microdialysis experiments to investigate the conversion of L-KYN or D-KYN to KYNA in the rat cerebellum *in vivo*. The enantiomers were infused for 2 h by reverse dialysis, and the content of KYNA was monitored in microdialysate samples for an additional 4 h. Perfusion with 100 μM L-KYN reversibly raised extracellular KYNA levels, reaching a maximum of 17.9 ± 3.7 times baseline levels 2 h after the beginning of the perfusion (Figure 4). Perfusion with 100 μM D-KYN produced a 10.7 ± 1.2-fold increase in extracellular KYNA levels after 2 h (Figure 5).

### ONOO^−^ Enhances KYNA Production *in vivo*

A brief (30 min) perfusion with 50 μM ONOO^−^ enhanced the concentration of extracellular KYNA, assessed by *in vivo* microdialysis in the cerebellum. This treatment raised KYNA levels, assessed in a single 30-min microdialysis fraction, from a basal value of 2.9 ± 0.3 nM to 11.4 ± 2.4 nM (*n* = 16; *P* < 0.01).

### ONOO^−^ Enhances KYNA Production from L-KYN and D-KYN *in vivo*

The 30-min pre-perfusion with ONOO^−^ substantially enhanced KYNA production from L-KYN or D-KYN (100 μM each) when the enantiomers were applied by reverse dialysis for 2 h immediately following the termination of perfusion with the pro-oxidant. In both cases, stimulation was greatest in the first 30 min and subsided gradually with time, probably indicating the waning influence of the discontinued perfusion with ONOO^−^ (Figures 4, 5). Peak potentiation, compared to control animals perfused without ONOO^−^ pre-treatment, was 4.1 ± 1.1-fold for L-KYN (Figure 4) and 3.2 ± 0.6-fold for D-KYN (Figure 5).

## Discussion

The present study demonstrated that KYNA can be synthesized enzymatically from both L-KYN and D-KYN in the rat cerebellum and, furthermore, that KYNA production from either enantiomer is enhanced in the presence of ROS. These results, which were first obtained *in vitro* and then confirmed *in vivo*, suggest that KYNA levels in the cerebellum can normally be controlled by several biosynthetic mechanisms. Conceivably, the relative significance of these biosynthetic routes may differ under various physiological conditions as well as in various pathological situations involving the cerebellum.

Irreversible transamination of L-KYN by KATs is considered the main means of KYNA formation in the mammalian brain (Turski et al., 1989) and was verified in the present study using the non-specific KAT inhibitor AOAA as an experimental tool. However, D-KYN, too, can serve as a substrate of KATs in both peripheral tissues and the brain (Pérez-de la Cruz et al., 2012), and this comparatively minor synthesis route was confirmed here using cerebellar tissue homogenates. Moreover, in contrast to L-KYN, D-KYN is an excellent substrate of DAAO, which is highly concentrated in the cerebellum (Horiike et al., 1994; Moreno et al., 1999; Verrall et al., 2007). We were therefore not surprised to observe that the cerebellar production of KYNA from D-KYN was quantitatively similar to KYNA formation from L-KYN both *in vitro* and *in vivo*, and that the three DAAO inhibitors BA, KA and MPC all caused a substantial reduction in KYNA synthesis from D-KYN.

Our study also showed that the redox environment has a substantial influence on KYNA production in the cerebellum since the pro-oxidant agents OH• and ONOO^−^ enhanced KYNA formation from either L-KYN or D-KYN in an artificial milieu *in vitro*. As the effect of ONOO^−^ exceeded the effect of OH•, this pro-oxidant was then tested in cerebellar tissue homogenate where it greatly potentiated the ability of both KYN enantiomers to synthesize KYNA. Notably, subsequent *in vivo* experiments revealed a substantial increase in extracellular KYNA within 30 min after reverse dialysis of ONOO^−^ alone, suggesting that a pro-oxidative environment also stimulates the conversion of *endogenous* tryptophan or KYN to KYNA (cf. Introduction). Tryptophan, through its metabolites indole-3-pyruvic acid and KYN, and the subsequent production of the anti-oxidant KYNA (Politi et al., 1991; Lugo-Huitrón et al., 2011b; Ugalde-Muniz et al., 2012), may therefore provide a defense mechanism against the detrimental effects of ROS in the brain (see below). Also of note in this context, L-KYN reduces chemiluminescence of luminol induced by H_2_O_2_ or chloramine (Weiss et al., 2013) and is able to inhibit ROS production by neutrophils (Genestet et al., 2014).

ROS and reactive nitrogen species (RNS) are produced during physiological processes and, by interacting with proteins, fatty acids and DNA, perform numerous roles in the regulation of cellular function (Dröge, 2002; Koskenkorva-Frank et al., 2013). Increased production of ROS and RNS and/or insufficient endogenous defense mechanisms in neurons or astrocytes can lead to functional impairments and cause cellular injury (Dringen et al., 2000; Valko et al., 2007; Scherz-Shouval and Elazar, 2011). Specifically, ONOO^−^ is a potent, short-lived oxidant species that is produced by the reaction of nitric oxide (NO•) and superoxide (O_2_•^−^). As NO• is a relatively stable and highly diffusible free radical (Szabó et al., 2007; Botti et al., 2010), the formation of ONOO^−^ is spatially associated with the sources of O_2_•^−^ (such as the mitochondrial respiratory complex). This allows ONOO^−^ to inhibit antioxidant enzymes or neutralize antioxidants (Ischiropoulos et al., 1992; Quijano et al., 1997; MacMillan-Crow et al., 1998; Aykaç-Toker et al., 2001; Savvides et al., 2002) and, consequently, to cause apoptotic or necrotic cell death (Szabó et al., 2007; Franco et al., 2013). Additionally, ONOO^−^ produces secondary reactive species such as nitrogen dioxide, hydroxyl and carbonate radicals, all of which interfere with a large number of cellular functions and increase cellular vulnerability (Radi et al., 1991; Bartesaghi et al., 2006). Interestingly, in the present study all three DAAO inhibitors attenuated KYNA production in the presence of ONOO^−^ *in vitro* to various degree (Figure 3), indicating that these compounds also have antioxidant activity (see also Gomes et al., 2001).

In the brain, dysregulated redox processes have been proposed to constitute a critical factor in the pathophysiology of neurodegenerative disorders and in major psychiatric diseases including schizophrenia and depressive disorders (Okusaga, 2013; Cahill-Smith and Li, 2014; Salim, 2014; Black et al., 2015; Gu et al., 2015). Notably, oxidative stress in the brain increases with advancing age (Tian et al., 1998), so that redox phenomena may also play a causative role in age-related structural and cognitive deficits (Dröge and Schipper, 2007; Brawek et al., 2010). As abnormal cerebral disposition of KYNA, too, has been linked to various brain pathologies (for review, see Schwarcz et al., 2012), the present findings raise the possibility that the boosting of brain KYNA levels by ROS and/or RNS may be functionally related to the pathological effects of the pro-oxidants. In other words, we speculate that the increased generation of KYNA in the presence of harmful free radicals may have evolved as a (neuro)protective mechanism to counter the effects of oxidative stress (Lugo-Huitrón et al., 2011a; Ugalde-Muniz et al., 2012). This increase in brain KYNA levels may also have detrimental consequences, however. Thus, even relatively modest elevations in brain KYNA cause a reduction in the extracellular concentrations of several classic neurotransmitters, including dopamine, glutamate and GABA (Carpenedo et al., 2001; Rassoulpour et al., 2005; Wu et al., 2010; Beggiato et al., 2014), and may therefore have adverse effects, especially on cognitive functions (Pocivavsek et al., 2011, 2012). This hypothesis, as well as the detailed cellular, subcellular and molecular mechanisms involved in the interactions between KYNA, ROS and RNS, is currently under investigation in our laboratories.

Our results also raise the question of a possible role of D-tryptophan or D-KYN in this context. Thus, whereas the biology of the essential amino acid L-tryptophan and its major catabolic product, L-KYN, in mammalian systems is reasonably well understood, information about possible roles of their respective D-enantiomers is still very sparse. In fact, D-KYN has so far not been identified as an endogenous constituent of mammalian tissues, though it is readily produced from D-tryptophan, which can originate from the microbial flora (Friedman, 1999; Rodríguez-Crespo, 2008), from the diet (Friedman, 2010) or, possibly, from enzymatic cleavage of D-tryptophan-containing polypeptides, which are found in several vertebrate species including humans (Jilek et al., 2005). Experimentally, D-KYN formation from D-tryptophan, and further degradation to KYNA, has been documented in several mammalian species and in several organs including the brain (Tashiro et al., 1961; Higuchi and Hayaishi, 1967; Loh and Berg, 1971; Ishii et al., 2010; Notarangelo et al., 2013). Importantly, in line with the high DAAO activity of the cerebellum (Horiike et al., 1994; Verrall et al., 2007), KYNA production from systemically applied D-tryptophan or D-KYN is especially pronounced in the cerebellum (Wang et al., 2012; Notarangelo et al., 2013). Oxidative processes in this brain region, which may play a substantive role in a considerable number of grave neurological and psychiatric diseases including cerebellar ataxia and autism (Chauhan and Chauhan, 2006; Kern and Jones, 2006; Wang et al., 2011; Goldani et al., 2014; Rossignol and Frye, 2014; Salim, 2014; Steullet et al., 2014), could therefore conceivably have etiological links to both L-KYN and D-KYN or their respective bioprecursors. These links could be especially relevant in situations involving dysfunctions of the immune system, since increased D-KYN formation from D-tryptophan, as well as enhanced L-KYN formation from L-tryptophan, is seen under inflammatory conditions due to a pronounced up-regulation of the non-stereospecific enzyme indoleamine-2, 3-dioxygenase (Johnson et al., 2009).

The fact that the cerebellum can produce KYNA by routes other than the canonical pathway has likely functional implications since both α7nAChR and NMDAR, which can serve as targets of KYNA (Kessler et al., 1989; Hilmas et al., 2001; Alkondon et al., 2011), are abundant in this area of the brain (Caruncho et al., 1997; Dumas, 2005; Llansola et al., 2005; Taslim and Saeed Dar, 2011). While early experiments, performed mostly using cultured cerebellar neurons, clearly documented inhibition of NMDAR function by high (millimolar) concentrations of KYNA (Gallo et al., 1987; Brockhaus and Deitmer, 2002), recent studies suggest that α7nAChRs may, in fact, be the preferential target of *endogenous* KYNA in the cerebellum. Thus, increases in cerebellar KYNA concentrations in the nanomolar range reduce extracellular glutamate levels locally *in vivo* and, as also seen in several regions of the forebrain (Albuquerque and Schwarcz, 2013), this effect can be duplicated by other α7nAChR—but not NMDAR—antagonists (Wu and Schwarcz, 2013 and unpublished data). Interestingly, and in line with the well-documented network connecting the cerebellum with the midbrain and the forebrain (Clower et al., 2005; Mittleman et al., 2008), even a moderate elevation of cerebellar KYNA levels controls the extracellular levels of glutamate and dopamine in the distant prefrontal cortex (Wu and Schwarcz, 2013). Such insights provide a conceptual framework for studies designed to explore functional links between (fluctuations in) cerebellar KYNA and motor, cognitive and other forebrain functions that, are influenced by the cerebellum (Ichinohe et al., 2000; Hoshi et al., 2005; Akkal et al., 2007).

**Figure 6:**
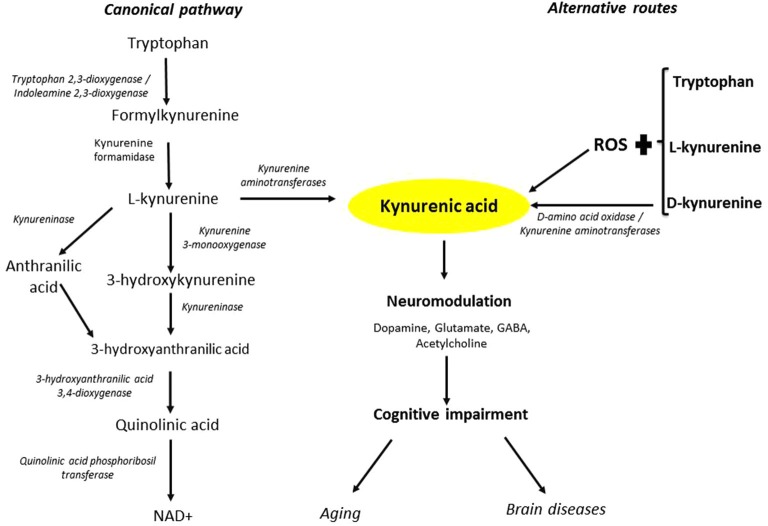
**Routes of KYNA formation**. The canonical pathway is initiated by the oxidative degradation of tryptophan to L-KYN and the subsequent irreversible transamination of L-KYN to KYNA. Alternative routes to KYNA involve interactions between tryptophan, L-KYN and D-KYN, respectively, and ROS. Moreover, DAAO can convert D-KYN to KYNA. Fluctuations in KYNA levels can alter glutamatergic, dopaminergic, GABAergic and cholinergic neurotransmission, and may thus be causally involved in cognitive impairments in aging and major brain diseases.

In summary, the present study demonstrates that mechanisms other than the classic enzymatic transamination of L-KYN, namely DAAO-catalyzed synthesis from D-KYN, and the interplay between L-KYN or D-KYN (and possibly L-tryptophan or D-tryptophan) with ROS, can contribute to the formation of KYNA in the rat cerebellum (Figure 6). These findings have ramifications for the role of KYNA in cerebellar physiology and pathophysiology and suggest novel strategies for normalizing impaired cerebral KYNA function in aging and major brain diseases.

## Conflict of Interest Statement

The authors declare that the research was conducted in the absence of any commercial or financial relationships that could be construed as a potential conflict of interest.

## Abbreviations

AOAA: Aminooxyacetic acid
D-KYN: D-Kynurenine
DAAO: D-amino acid oxidase
DMSO: Dimethylsulfoxide
D-Trp: D-tryptophan
3-HK: 3-Hydroxykynurenine
L-KYN: L-Kynurenine
KYNA: Kynurenic acid
KP: Kynurenine pathway
KATs: Kynurenine aminotransferases
MPC: 3-Methylpyrazole-5-carboxylic acid
NMDA: N-methyl-D-aspartate
ONOO^−^: peroxynitrite
OH•: Hydroxyl radical
PFC: Prefrontal cortex
ROS: Reactive oxygen species.
